# Validation and characterization of *Citrus sinensis* microRNAs and their target genes

**DOI:** 10.1186/1756-0500-5-235

**Published:** 2012-05-15

**Authors:** Changnian Song, Mingliang Yu, Jian Han, Chen Wang, Hong Liu, Yanping Zhang, Jinggui Fang

**Affiliations:** 1 College of Horticulture, Nanjing Agricultural University, 1 Weigang, Nanjing, 210095, China; 2 Institute of Horticulture, Jiangsu Academy of Agricultural Sciences, Nanjing, 210014, China

## Abstract

**Background:**

MicroRNAs play vital role in plant growth and development by changeable expression of their target genes with most plant microRNAs having perfect or near-perfect complementarities with their target genes but miRNAs in *Citrus sinensis* (csi-miRNAs) and their function have not been widely studied.

**Findings:**

In this study, 15 potential microRNAs in *Citrus sinensis* (csi-miRNAs) were identified and bioinformatically validated using miR-RACE, a newly developed method for determination of miRNAs prediction computationally. The expression of these fifteen *C. sinensis* miRNAs can be detected in leaves, stems, flowers and fruits of *C. sinensis* by QRT-PCR with some of them showed tissue-specific expression. Six potential target genes were identified for six csi-miRNAs and also experimentally verified by Poly (A) polymerase -mediated 3′ rapid amplification of cDNA ends (PPM-RACE) and RNA ligase-mediated 5′ rapid amplification of cDNA ends (RLM-RACE) which mapped the cleavage site of target mRNAs and detected expression patterns of cleaved fragments that indicate the regulatory function of the miRNAs on their target genes.

**Conclusions:**

Our results confirm that small RNA-mediated regulation whereby all csi-miRNAs regulate their target genes by degradation.

## **Findings**

### Background

MicroRNAs (miRNAs) are small, single-stranded, non-protein coding RNAs of ~21 nt in length, present in plants and animals which regulating the gene expression at the posttranscriptional levels by binding to target mRNAs for mRNA cleavage or inhibition of mRNA translation. miRNA play an important role in the plant kingdom are evolutionarily conserved from mosses and ferns to higher flowering plants. This conservation has been used as a powerful strategy for identification or prediction of miRNAs by homology searches in other species [1,2]. Sunkar et al [3] reported that 682 miRNAs have been identified in 155 diverse plant species and they found more than 15 conserved miRNA families in eleven 11 plant species by searching all publicly available nucleotide databases. In prior studies, combination of computational prediction and experimental verification was used to identify miRNAs, with experimental validation mainly focused on determining the expression of miRNAs using robust techniques of RNA blotting and/or RT-PCR. However, these two techniques can only confirm existence and size, but not the full precise sequences, especially termini nucleotides computationally predicted miRNAs [4].

In addition to the economic value of citrus, availability of a large number of expressed sequence tags (ESTs) in citrus makes it an excellent source of experimental material for elucidation of gene expression and regulation. miRNAs have been extensively studied in the modern research but systematic study has not been performed on leaf, stem, flower, and fruit growth and development of *C. sinensis.*. Recently, three study groups identified several miRNAs from citrus using computational approaches and deep sequencing [1,3,5,6], but the number of *C. sinensis* miRNAs predicted still remains low, and experimental validation hardly carried out. In this study, precise sequences, especially terminal nucleotides of 15 csi-miRNAs were validated by miR-RACE, a recent technique developed by Song *et al*. [4] based on 17 miRNAs from 13 miRNA families that were computationally predicted in our previous study. The ubiquitous expression of these 15 miRNAs can be detected in different tissue of citrus by QRT-PCR. For detection of miRNA-mediated cleavage products, and determination of the manner of miRNA regulation on target genes, Poly (A) polymerase -mediated 3′ rapid amplification of cDNA ends (PPM-RACE) and RNA ligase-mediated 5′ rapid amplification of cDNA ends (RLM-RACE) developed. The result suggested that the importance of miRNAs in regulating development of *C. sinensis* and indicate that comprehensive studies of miRNAs in citrus would facilitate further understanding of regulatory mechanisms behind floral induction, stage transition and organ genesis. The new strategy developed here can definitely facilitate ease of studying the mechanisms of miRNA regulation on their target genes.

## Results

### Precise sequence validation of csi-miRNAs

In our study we have detected the new 17 csi-miRNAs [7], belonging to be 13 families from *C. sinensis* genome by computational screening and it is the first time being reported in China. With conserved complementarities to miRNAs in other plant species and one important member of each family were successfully verified. The difference between our method and traditional RACE lies in the gene-specific primers used (Additional file 1). The PCR products were cloned and sequenced when they yielded reliable band. Sequences of each pair of 5′ and 3′ PCR products in each of the 15 csi-miRNAs were spliced to generate whole mature miRNA sequences (Additional file 2).

In summary, sequencing results were also used to confirm the predicted seventeen csi-miRNAs and to identify their precise end sequences, in which of them this study identified fifteen csi-miRNAs (csi-miR160, csi-miR162, csi-miR165, csi-miR166a, csi-miR166b, csi-miR172, csi-miR390, csi-miR482a.2, csi-miR482a.4, csi-miR530, csi-miR844, csi-miR950, csi-miR1027, csi-miR1044-3p, and csi-miR1426, Table 1). Along with demonstrate of four conserved miRNAs (csi-miR160, csi-miR162, csi-miR165, and csi-miR390) and the sequence were identical both in length and nucleotide to their orthologs in *Arabidopsis* or other plants (Additional file 3), and the other 11 non-conserved miRNAs sequence varied at both internal and terminal nucleotides.

**Table 1:** **List of computer predicted and verified miroRNA in *C. sinensis* **

**miroRNA**	**Predicted miRNA sequence**	**Verified miRNA sequence**
csi-miR160a	UGCCUGGCUCCCUGUAUGCCA	UGCCUGGCUCCCUGUAUGCCA
csi-miR162	UCGAUAAACCUCUGCAUCCAG	UCGAUAAACCUCUGCAUCCAG
csi-miR165a	UCGGACCAGGCUUCAUCCCCC	UCGGACCAGGCUUCAUCCCCC
csi-miR166a	UCGGACCAGGCUUCAUUCCCCC	UCGGACCAGGCUUCAUUCCCCC
csi-miR166b	UCGGACCAGGCUUCAUUCCCG	UCGGACCAGGCUUCAUUCCCG
csi-miR172a	AGAAUCUUGAUGAUGCUGCAA	AGAAUCUUGAUGAUGCUGCAA
csi-miR390	AAGCUCAGGAGGGAUAGCGCC	AAGCUCAGGAGGGAUAGCGCC
csi-miR482a.1	UCUUCCCUACUCCACCCAUG	
csi-miR482a.2	UCUUCCCUACUCCACCCAUGCC	UCUUCCCUACUCCACCCAUGCC
csi-miR482a.3	UCUUCCCUACUCCCCCCAUG	
csi-miR482a.4	UCUUCCCUACUCCCCCCAUGCC	UCUUCCCUACUCCCCCCAUGCC
csi-miR530	UGCAUUUGCAGGUGCAUCAU	UGCAUUUGCAGGUGCAUCAU
csi-miR844	CUAUAAGCCAUCUCACUAGGU	CUAUAAGCCAUCUCACUAGGU
csi-miR950	UCAGGUCCUCAGUGGUCCAU	UCAGGUCCUCAGUGGUCCAU
csi-miR1027	UUUCUAUCAUCUAUUCCAAUG	UUUCUAUCAUCUAUUCCAAUG
csi-miR1044-3p	UUGUAGUGCGUAUUGGUAUU	UUGUAGUGCGUAUUGGUAUU
csi-miR1426	UGAAUCUUGAUGAUGAUUGAU	UGAAUCUUGAUGAUGAUUGAU

### Expression analysis of csi-miRNAs by QRT-PCR

**Figure 1:**
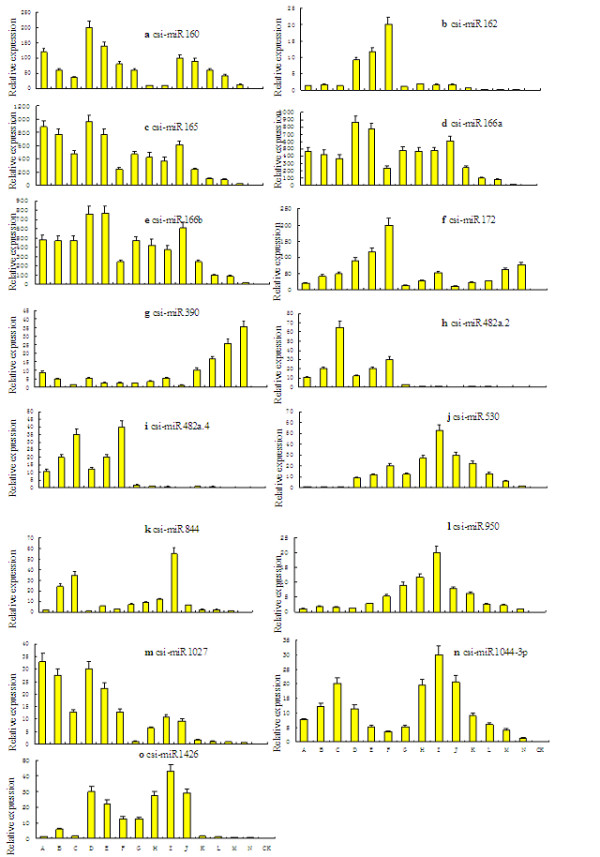
**Expression patterns of fifteen miRNA predicted from *C. sinensis* .** QRT-PCR of total RNA isolated from tissues at different stages of development*.* A, B, C, D, E, F, G, H, I, J, K, L, M, and N are samples of young stems, mature stems, old stems, young leaves, mature leaves, old leaves, flower buds, half open flowers, open flowers, and fruits of different stages (15, 45, 75, 105 and 145 DAFB), respectively. Each reaction was repeated three times and the template amount was corrected by 5.8 s rRNAs.

**Figure 2:**
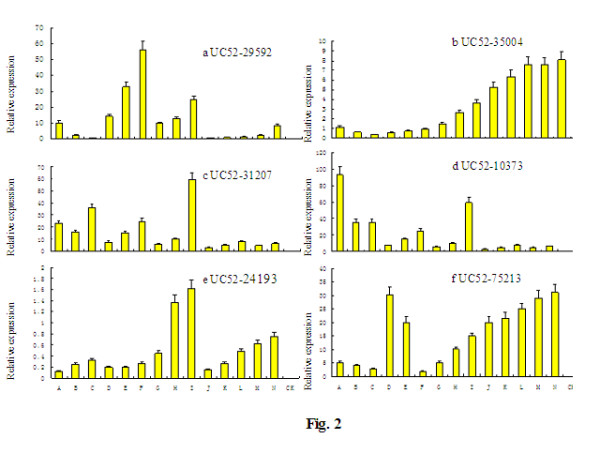
**Mapping of the mRNA cleavage sites by PPM-RACE and RLM-RACE.** Each top strand (black) depicts a miRNA complementary site, and each bottom strand depicts the miRNA (red). Watson-Crick pairing (vertical dashes) and G: U wobble pairing (circles) are indicated. The arrows indicate the 5′ termini of mRNA fragments isolated from citrus, as identified by cloned PPM-RACE and RLM-RACE products, with the frequency of clones shown. Only the cloned sequences that matched the correct gene and had 5′ ends within a 100 nt window centered on the miRNA validation are included. The miRNA sequence shown indicated in lower case corresponds to the most common miRNA supported by the miRNA PCR (1 out of 4 PCR clones). PPM-RACE and RLM-RACE were used to map the cleavage sites. The partial mRNA sequences from the target genes were aligned with the miRNAs. The numbers indicate the fraction of cloned PCR products terminating at different positions.

Preferential expression of 15 csi-miRNAs was also carried out for a more comprehensive and efficient characterizations of the miRNAs in the organism along with provide clues on their physiological functions. Thus we can claim that QRT-PCR [8] is a reliable method for detecting and measuring the expression levels of 15 csi-miRNAs and detection and measurement of cis-miRNA expression levels is one of the most important works in the field of horticulture carried out in miRNA studies in citrus. We examined their expression by analyzing total RNA samples from various tissues of sweet orange tree using QRT-PCR (Figure 1). Majority were expressed ubiquitously in all tissues, while some were expressed in tissue-, and/or growth-stage-specific patterns, with the expression patterns of these cis-miRNAs being grouped into several situations. Expression of csi-miR160, csi-miR165, csi-miR166a, and csi-miR166b were strong in young and old leaves, high in young, mature and old stems, flower buds, half open flowers, fully open flowers and fruits (before 45 DAFB), and low in fruits at 45 DAFB (Figure 1a1c1d, and 1e). Although all tissue showed some minor organ specificity; expression in young organs seemed stronger than older tissues. Some miRNAs might display species-specific and/or developmental stage-specific expression patterns, and the best example were observed in csi-miR162, csi-miR390, csi-miR482a.2 and csi-miR482a.4 (Figures 1b, 2f,2g and 2h), all of which had preferential expression in leaves and stems of sweet orange but almost no expression in flowers and fruits. csi-miR162 has superior expression in young, mature and old leaves, and displayed low expression in other tissues (Figure 1b), while csi-miR390 has strong expression in fruits at different stages but displayed low or no expression in other tissues (Figure 1f). This was also observed for csi-miR482a.2 and csi-miR482a.4 (Figure 1f and 1g), both of which had better expression in leaves and stems but almost no expression in flowers and fruits. csi-miR172 was highly expressed in old stems, old leaves, flowers, and fruits at 145 DAFB, and has low or no expression in other tissues (Figure 1e). csi-miR530, csi-miR844, csi-miR950, csi-miR1027, csi-miR1044-3p and csi-miR1426 showed lower expression in all tested tissues (Figure 1i1j1k1l1m and 1n) than the above csi-miRNAs. csi-miR530 and csi-miR950 were highly expressed in flower buds and fruits at 15 DAFB, and has moderate or much weaker expression in all the other tissues (Figure 1i and 1k). csi-miR844 showed expression in all tissues, with strong expression in fully open flowers and mature, and old stems (Figure 1j). Expression of csi-1027 was strong in young and old leaves and high in young, mature and old stems, flower buds, half open flowers, fully open flowers and fruits (before 45 DAFB), while it was low in fruits at 45 DAFB (Figure 1l). csi-miR1044-3p seemed to be expressed in all tested sweet orange tissue, with expression in old stems, young leaves, open flowers, and fruits at 15 DAFB being high and moderate or much weaker in all the other tissues (Figure 1m). csi-miR1426 has preferential expression in different development stages of leaves and flowers, but has low or no expression in other tissues (Figure 1n).

In summary, 15 potential *C. sinensis* miRNAs, all identified by miR-RACE were validated by QRT-PCR with some being expressed ubiquitously in all tissues with tissue and/or growth stage specific characteristics reflected at different expression levels. This can be an essential reference for comprehensive analysis of the function of these csi-miRNAs.

### Potential target prediction for fifteen *C. sinensis* miRNA

The potential miRNA target genes of several conserved *C. sinensis* miRNAs, such as UC52-29592 (Auxin response factor 10, ARF10) for miR160, UC52-35004, UC52-31207, and UC52-10373 (Homeo domain leucine zipper, HD-Zip protein) for miR165, UC52-31207, and UC52-10373 (HD-Zip protein) for miR166, UC52-24193 (AP2) for miR172 (Additional file 4: Table S3). No targets were found in *C. sinensis* for csi-miR162, csi-miR530, csi-miR844, csi-miR950, csi-miR1027, csi-miR1044-3p, and csi-miR1426but important targets included *NB-LRR* disease resistance gene analogs, such as UC52-75213 that was similar to *P. trichocarpa* XM_002298664 cc-nbs-lrr resistance protein (Additional file 4).

### miRNA target gene expression analysis

For advance studies on target mRNAs and miRNA expression at different plant development stages and organs, QRT-PCR analysis was done to detect these target mRNAs in the sampled sweet orange organs. All the target mRNAs were expressed ubiquitously in all tissues with tissue- and/or growth-stage-specific characteristics reflected from different expression levels by QRT-PCR. The detection of miRNA target genes and expression of the corresponding miRNA shows mutual growth and decline trends in expression (Figure 2) indicating that the six target genes can be actively cleaved by miRNAs. Our result indicated that the expressions of miRNAs and of their target genes are generally negatively correlated.

### Identification of miRNA-guided cleavage of target mRNAs in *C. sinensis* miRNAs

**Figure 3:**
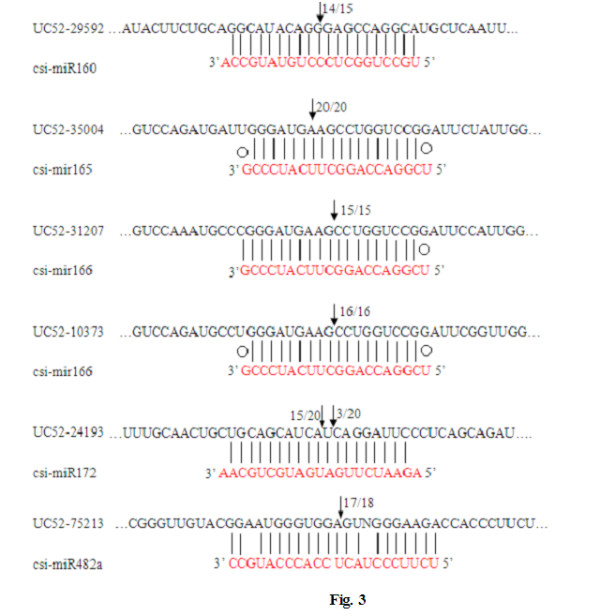
**Relative expression levels of *C. sinensis* miRNA target mRNAs.** QRT-PCR of total RNA isolated from tissues at different development stages*.* A, B, C, D, E, F, G, H, I, J, K, L, M, and N are the samples of young stems, mature stems, old stems, young leaves, mature leaves, old leaves, flower buds, half open flowers, open flowers, and fruits of different stages (15, 45, 75, 105 and 145 DAFB) respectively. Each reaction was repeated three times and the template amount was corrected by 5.8 s rRNAs.

To verify the nature of csi-miRNA targets and to study regulation of csi-miRNAs on their target genes, PPM-RACE and RLM-RACE experiments was done for better characterization of the csi-miRNAs predicted [7]. All six csi-miRNAs guided target cleavage, most often at the length nucleotide, as expected (Figure 3) with the six predicted targets having specific cleavage sites corresponding to the miRNA complementary sequences and might be regulated by the miRNAs in the style of small interfering RNAs (siRNAs) [9] directing the cleavage of mRNA targets with extensive complementarity to the miRNAs [10].

### Detection of miRNA cleaved target mRNAs by PPM-RACE, RLM- RACE

miRNAs regulate their target genes as well as the degradation levels of the target mRNAs, we first employed a new and efficient strategy that integrates preparation of cleaved target miRNAs, QRT-PCR amplification of 3′ and 5′ end fragments of the cleaved products, sequence-directed cloning, and alignment analysis (Additional file 5). Even though the quantity of the 5′ ends has been reported to be lower than that of the corresponding 3′ ends of the phase [11], and despite the 5′ and 3′ ends of the target gene theoretically showing relatively consistent trends, it was necessary to know the information of both the 3′ and 5′ ends from the cleaved mRNA for comprehensive understanding of regulation of miRNAs on their target genes. The existing levels of six group cleaved by the six miRNAs exhibited similar patterns of expression (Additional file 5), with their corresponding miRNAs. Majority of the miRNA cleaved target mRNAs existed ubiquitously in all tissues, and some showed tissue-, and/or growth-stage-specific expression patterns.

## Discussion

Although plant miRNAs have been studied in the recent past, but few studies have been conducted on *C. sinensis*, a most important fruits crops in the world. Sunkar and Jagadeeswaran [3] identified several miRNAs from citrus EST by bioinformatics, but *C. sinensis* miRNAs still remain largely unknown and few the identified *C. sinensis* miRNAs have not yet been experimentally confirmed. To elucidate the functions and the regulation pathways of miRNAs in citrus, it is important to know the localization of specific miRNAs. Although a numbers of methods have been developed for computational prediction of miRNAs, their disadvantages have not been overcome experimentally. These disadvantages include the aspect whereby precise sequences of miRNAs are seldom determined and where several candidate miRNA orthologs or paralogs might be predicted for a specific miRNA (e.g. the prediction of csi-miR482). We report the use of miR-RACE as the first experimental approach to overcome this problem, through employment of challenging steps.

Reverse transcription followed by quantitative PCR analysis (QRT-PCR) with nonspecific double-stranded DNA-binding fluorophores, such as SYBR Green, is a powerful alternative for highly sensitive, rapid, multi-parallel, and cost-effective expression analysis [12]. Shi and Chiang [13] and Chen *et al*. [8] reported two QRT-PCR-based methods to measure the levels of mature miRs. The first approach relies on in vitro polyadenylation of mature miRs followed by RT with an oligo(dT) adapter primer and amplification using SYBR Green with a miR-specific forward primer and a compatible reverse primer. In the second approach, each specific miR is reverse transcribed from total RNA using a specific stem-loop primer, followed by TaqMan PCR amplification. Although it is desirable to quantify the biologically active mature miR species, a limitation of both QRT-PCR methods is that they are unable to differentiate the expression strengths of *MIRNA* genes that yield (nearly) identical mature miR molecules. Varkonyi-Gasic *et al*. [14] describe and provide protocols for an end-point and real-time looped RT-PCR procedure. This method enables fast, sensitive and specific miRNA expression profiling and is suitable for facilitation of high throughput detection and quantification of miRNA expression.

Although miRNAs generally function as negative regulators of gene expression by mediating the cleavage of target mRNAs [15] or by repressing their translation [16], the cleavage of target mRNAs appears to be predominant mode in gene regulation by plant miRNAs [10]. Finding the cleavage site supposedly located in the sequence complementary to the miRNA in the target gene is essential for verification of the cleavage of target genes. Among the methods used to observe miRNA-dependent cleavage of targets, RLM-RACE and PARE is the most useful. PARE using a second sequencing technology (e.g. Illumina-Solexa) is another new approach being adopted for the analysis of miRNAs targets [17-20]. The high number of sequence reads promises sensitivity, yet the necessary expertise required and the labor and cost involved are considerable. In this study, we developed a combination of PPM-RACE and RLM-RACE for mapping miRNA-mediated cleavage products and validation of miRNA targets, which takes advantage of modified 5′ rapid and 3′ rapid amplification of cDNA ends as well as QRT-PCR and bioinformatic tools. The PPM-RACE and RLM-RACE was done on unigenes for detection and cloning of the mRNA fragments which correspond precisely to the predicted products of miRNA processing.

We performed the PPM-RACE and RLM-RACE on unigenes so as to detect and clone the mRNA fragments corresponding precisely to the predicted products of miRNA processing. The findings from this PPM-RACE and RLM-RACE could be used as a criterion for confirming the putative targets. In total, we performed PPM-RACE and RLM-RACE assays on six predicted target genes (UC52-29592, UC52-35004, UC52-31207, UC52-10373, UC52-24193, and UC52-75213) which are representative targets of five conserved miRNAs (miR160, miR165, miR166, miR172, and miR482a, respectively). Unigene C46-29592 comprises of auxin response factors (ARFs) that bind to auxin response elements in promoters of early auxin response genes [21]. UC52-35004, UC52-31207, and UC52-10373 were all conversed Class-III homeodomain leucine zipper (HD-ZIP) proteins. Another experimentally confirmed miRNA-targeted transcription factor is *APETALA2* (*AP2*), which controls floral development and phase transition in Arabidopsis [15]. UC52-10373 is similar to Arabidopsis proteins coded by IRX12 copper ion binding/ oxidoreductase (IRX12CBO), which coded for a protein highly homologous to NB-LRR disease resistance protein. Targeting of NB-LRR genes by miRNAs has previously been reported in poplar, Arabidopsis and grape [22-24], but its contribution to disease resistance is still poorly characterized. In this research, UC52-24193, which is predicted target of miR172, was found to be a member of the *AP2* gene family. All four predicted targets were found to have specific cleavage sites corresponding to the miRNA complementary sequences (Figure 3), and the most common 5′ end of the mRNA fragments mapped to the nucleotides that pair with the 10th miRNA nucleotide from the 5′ ends. miRNAs may directly target transcription factors that affect plant development and also specific genes that control metabolism. In our study, it appears that our predicted targets play role not only in development, but also in diverse physiological processes. In summary, the efficient and powerful approach developed herein can be successfully used to validate the expression of csi-miRNA cleaved target mRNAs.

## Conclusion

This is the first time we discovered precise sequences of fifteen csi-miRNAs through miR-RACE. In addition, expression of these 15 *C. sinensis* miRNAs can be detected by QRT-PCR in young, mature and old leaves, young, mature and old stems, flower buds, half open flowers, open flowers, and developing fruits at 15, 45, 75, 105 and 145 DAFB, along with csi-miRNAs showing tissue-specific expression. And six potential target genes for six csi-miRNAs were experimentally verified by PPM-RAC and RLM-RACE. These results showed that regulatory miRNAs exist in agronomically *C. sinensis* and play an essential role in citrus growth, development, and response to disease.

## Materials and methods

### Plant materials

Samples were collected from two five-year-old ‘Newhall’ navel oranges (*Citrus sinensis* [L.] Osbeck) trees grown at the Suzhou Evergreen Fruit Tree Research Institute, China. Young, mature and old leaves and stems, flower buds, half open and fully open flowers and fruits at different stages of development (15, 45, 75, 105 and 145 days after full bloom, DAFB) were collected. After collection, all samples were immediately frozen in liquid nitrogen and stored at −80°C.

### *C. sinensis* miRNAs and prediction of potential target mRNAs

To validate bioinformatically predicted miRNAs, 17 mature miRNA sequences (csi-miR160, csi-miR162, csi-miR165, csi-miR166a, csi-miR166b, csi-miR172, csi-miR390, csi-miR482a.1, csi-miR482a.2, csi-miR482a.3, csi-miR482a.4, csi-miR530, csi-miR844, csi-miR950, csi-miR1027, csi-miR1044-3p, and csi-miR1426) were downloaded from the miRNA Registry database (Release 15.0, May 2010; http://microrna.sanger.ac.uk) and from previous publications [7].

Putative *C. sinensis* miRNAs were blasted against Harvest C52 Citrus unigene database on the Harvest Blast Search web server (http://138.23.191.145/blast/index.html). BLASTn hits with less than four nucleotide mismatches (plus/minus) were chosen as candidate targets, and were then searched in the Citrus Harvest 1.20 program using BLASTx to obtain their putative functions. Meanwhile, citrus miRNAs were blasted with citrus ESTs by Blast 2.17 (Elue set as 10). ESTs with less than four nucleotide mismatches (plus/minus) were extracted and then annotated by online BLASTx search against SwissProt protein sequences (SwissProt) or nondundant protein sequence (nr) database on the NCBI web server.

### Low molecular weight RNA extraction and construction of small RNA cDNA libraries

Total RNA isolated from 100 mg of previously collected tissue using TRIZOL reagent (Invitrogen, Life Technologies, Carlsbad, CA). Low and high molecular weight RNAs (LMW and HMW RNA) were separated with 4 M LiCl [2,24]. The procedure of construction of small RNA cDNA libraries as used by Fu *et al*. [25] was followed to generate the miRNA-enriched library.

### Verification of csi-miRNAs precise sequence by miR-RACE

In validation of csi-miRNAs using miR-RACE [4], cDNA libraries from the small RNA pool of RNA samples isolated from various organs and tissues was amplified with mirRacer 5′ primer (5′-GGACACTGACATGGACTGAAGGAGTA-3′) and mirRacer 3′ primer (5′-ATTCTAGAGGCCGAGGCGGCCGACATG-3′) to generate a pool of non-gene-specific products. GSP1 and GSP2 were complementary to seventeen nucleotide length sequences of potential csi-miRNAs and a part of Poly (T) and 5′ adaptor (Additional file 1). In each case, a unique gene-specific DNA fragment was amplified. 5′ and 3′ end clones with PCR products of about 56 bp and 87 bp in length, respectively, were sequenced [4].

### Real-Time PCR of *C. sinensis* miRNAs and their target mRNAs

To amplify csi-miRNAs from the reverse transcribed cDNAs, we used precise sequences of the csi-miRNAs as the forward primers (Additional file 1) and the mirRacer 3′ Primer as the reverse primer [4]. RT-PCR was conducted with Rotor-Gene 3000 (Corbett Robotics, Australia) and the Rotor-Gene software version 6.1 [26]. Comparative quantification was used to determine relative expression levels as previously described [27]. 5.8 S rRNA used as a reference gene in the qPCR detection of miRNAs like in *Arabidopsis*[12] and data analyzed with an R^2^ above 0.998 using LinRegPCR program [28] and expression of experimentally determined and predicted target genes assayed by QRT-PCR as previously described by Wilson *et al*. [27]. The reverse transcription product was amplified using gene-specific primers that overlapped known or predicted cleavage sites (Additional file 6). Reactions were performed in triplicate on a Rotor-Gene 3000 (Corbett Robotics, Australia). Data was normalized to AT5G08290 [29] and analyzed using a comparative quantification procedure [26].

### Mapping of mRNA cleavage sites with PPM-RACE and RLM-RACE

To map miRNA-mediated cleavage products, and to determine the manner by which the miRNAs regulate their target genes, the new strategy developed in this study and appropriately named PPM-RACE and RLM-RACE, was used. The method comprises of the following main steps: HMW RNAs polyadenylated at 37°C for 60 min in a 50 μl reaction mixture with 5 μg of HMW RNAs, 1 mM ATP, 2.5 mM MgCl_2_, and 8 U poly (A) polymerase (Ambion, Austin, TX), and were ligated to a 5′ adapter (5′-CGACUGGAGCACGAGGACACUGACAUGGACUGAAGGA GUAGAAA-3′) using T4 RNA ligase (Invitrogen, Carlsbad, CA), respectively. Poly (A)-tailed HMW RNA recovered by phenol/chloroform extraction and ethanol precipitation. Poly (A)-tailed HMW RNA and adapter-ligated HMW RNA was recovered by phenol/chloroform extraction followed by ethanol precipitation. Reverse transcription was performed as done by Fu *et al.*[25]. PPM-RACE and RLM-RACE amplifications were performed according to the GeneRacer kit guide (Invitrogen). Gene-specific PPM-RACE and RLM-RACE reactions were performed with the GeneRacer Nested Primer and gene-specific primers as shown in Additional file 7 and Additional file 8. Amplification products were gel purified, cloned, and sequenced, and at least fifteen independent clones were sequenced.

## Competing interests

The authors declare that they have no competing interests.

## Authors’ contributions

SC and YM carried out the laboratory work and participated in manuscript draft writing. HJ performed bioinformatics analyses. LH and ZY participated in the design and coordination the study. WC constructed the sRNA library. FJ conceived, designed the study and revised this paper. All authors read and approved the final manuscript.

## Supplementary Material

Additional file 1 Primers used for miR-5′ RACE, miR-3′ RACE, and QRT-PCR of csi-miRNAs. Click here for file

Additional file 2 **3′ RACE and 5′ RACE products of csi-miRNAs amplified by PCR shown in an ethidium bromide-stained agarose gel.** The sizes of the molecular weight markers of the bottom and the second bottom bands are 50 bp and 100 bp, respectively. A: Lanes 1–15 are 5′ RACE products of csi-miR160, csi-miR162, csi-miR165, csi-miR166a, csi-miR166b, csi-miR172, csi-miR390, csi-miR482a.2, csi-miR482a.4, csi-miR530, csi-miR844, csi-miR950, csi-miR1027, csi-miR1044-3p, and csi-miR1426, respectively, while lanes 16–30 are their 3′RACE products. Click here for file

Additional file 3 **Alignment between csi-miRNAs and their orthologs in *Arabidopsis* or other plants. ** Click here for file

Additional file 4 **Predicted targets for fifteen miRNAs identified in *C. sinensis* . ** Click here for file

Additional file 5 **Expression patterns of 3′ (A) and 5′ (B) products of miRNA cleaved target genes from *C. sinensis* by RLM-RACE and PPM-RACE.** QRT-PCR of HMW RNA isolated from tissues at different development stages*.* A, B, C, D, E, F, G, H, I, J, K, L, M, and N are the samples of young stems, mature stems, old stems, young leaves, mature leaves, old leaves, flower buds, half open flowers, open flowers, and fruits of different stages (15, 45, 75, 105 and 145 DAFB) Each reaction was repeated three times and the template amount was corrected by 5.8 s rRNAs. Click here for file

Additional file 6 The primer sequences of miRNA target genes for QRT-PCR. Click here for file

Additional file 7 The primer sequences of the 5′ products of miRNA cleaved target genes for QRT-PCR. Click here for file

Additional file 8 Primer sequences of the 3′ products of miRNA cleaved target genes for QRT-PCR. Click here for file
