# Supplementary material for: Validation and characterization of Citrus sinensis microRNAs and their target genes
Source: BMC Res Notes. 2012 May 15;5:235. doi: 10.1186/1756-0500-5-235 (PMC3436860; doi:10.1186/1756-0500-5-235)
Supplement: Additional file 1 — Primers used for miR-5′ RACE, miR-3′ RACE, and QRT-PCR of csi-miRNAs. [file 1756-0500-5-235-S1.doc]

**Additional file 1 Primers used for miR-5’ RACE, miR-3’ RACE, and real-time PCR** of csi-miRNAs.

| miRNAs | GSP1 (5'→ 3') | GSP2 (5'→3') | GSP3 (5'→3') |
| --- | --- | --- | --- |
| csi-miR160a | TTTTTTTTTTTGGCATACAGGGAGCCAG | GGAGTAGAAATGCCTGGCTCCCTGTAT | TGCCTGGCTCCCTGTATGCCA |
| csi-miR162 | TTTTTTTTTCTGGATGCAGAGGTTTA | GGAGTAGAAATCGATAAACCTCTGCAT | TCGATAAACCTCTGCATCCAG |
| csi-miR165 | TTTTTTTTTTGGGGGATGAAGCCTGGT | GGAGTAGAAATCGGACCAGGCTTCATC | TCGGACCAGGCTTCATCCCCC |
| csi-miR166a | TTTTTTTTTTGGGGGAATGAAGCCTGG | GGAGTAGAAATCGGACCAGGCTTCATTC | TCGGACCAGGCTTCATTCCCCC |
| csi-miR166b | TTTTTTTTTTCGGGAATGAAGCCTGGT | GGAGTAGAAATCGGACCAGGCTTCATT | TCGGACCAGGCTTCATTCCCG |
| csi-miR172a | TTTTTTTTTTTTGCAGCATCATCAAGA | GGAGTAGAAAAGAATCTTGATGATGCT | AGAATCTTGATGATGCTGCAA |
| csi-miR390 | TTTTTTTTTTGGCGCTATCCCTCCTGA | GGAGTAGAAAAAGCTCAGGAGGGATAG | AAGCTCAGGAGGGATAGCGCC |
| csi-miR482a.2 | TTTTTTTTTTGGCATGGGTGGAGTAGG | GGAGTAGAAATCTTCCCTACTCCACCC | TCTTCCCTACTCCACCCATGCC |
| csi-miR482a.4 | TTTTTTTTTTGGCATGGGGGGAGTAGG | GGAGTAGAAATCTTCCCTACTCCCCCCAT | TCTTCCCTACTCCCCCCATGCC |
| csi-miR530 | TTTTTTTTTTATGATGCACCTGCAAAT | GGAGTAGAAATGCATTTGCAGGTGCAT | TGCATTTGCAGGTGCATCAT |
| csi-miR844 | TTTTTTTTTTACCTAGTGAGATGGCTT | GGAGTAGAAACTATAAGCCATCTCACT | CTATAAGCCATCTCACTAGGT |
| csi-miR950 | TTTTTTTTTTATGGACCACTGAGGACC | GGAGTAGAAATCAGGTCCTCAGTGGTC | TCAGGTCCTCAGTGGTCCAT |
| csi-miR1027 | TTTTTTTTTTCATTGGAATAGATGATAG | GGAGTAGAAATTTCTATCATCTATTCC | TTTCTATCATCTATTCCAATG |
| csi-miR1044-3p | TTTTTTTTTTAATACCAATACGCACTA | GGAGTAGAAATTGTAGTGCGTATTGGT | TTGTAGTGCGTATTGGTATT |
| csi-miR1426 | TTTTTTTTTTATCAATCATCATCAAGA | GGAGTAGAAATGAATCTTGATGATGATT | TGAATCTTGATGATGATTGAT |

GSP1 is the specific primer for miR-5’ RACE, and the underlined region base pairs with the 3’ poly(A)n; GSP2 is the specific primer used for miR-3’ RACE, and the underlined region base pairs with the 5’ adaptor; GSP3 is the specific primer used for miRNA quantitative real-time PCR (qRT-PCR).
