# Supplementary material for: Validation and characterization of Citrus sinensis microRNAs and their target genes
Source: BMC Res Notes. 2012 May 15;5:235. doi: 10.1186/1756-0500-5-235 (PMC3436860; doi:10.1186/1756-0500-5-235)
Supplement: Additional file 3 — Alignment between csi-miRNAs and their orthologs in Arabidopsis or other plants. [file 1756-0500-5-235-S3.doc]

**Table S2**

Alignment between csi-miRNAs and their orthologs in *Arabidopsis* or other plants.

| miRNA (5'→3') | Nucleotide order | | | | | | | | | | | | | | | | | | | | | | | | |
| --- | --- | --- | --- | --- | --- | --- | --- | --- | --- | --- | --- | --- | --- | --- | --- | --- | --- | --- | --- | --- | --- | --- | --- | --- | --- |
|  | 1 | 2 | 3 | 4 | 5 | 6 | 7 | 8 | 9 | 10 | 11 | 12 | 13 | 14 | 15 | 16 | 17 | | 18 | 19 | 20 | 21 | 22 | 23 | 24 |
| mir160 | U | G | C | C | U | G | G | C | U | C | C | C | U | G | U | A | U | | G | C | C | A |  |  |  |
| csi-mir160a |  |  |  |  |  |  |  |  |  |  |  |  |  |  |  |  |  | |  |  |  |  |  |  |  |
| mir162 | U | C | G | A | U | A | A | A | C | C | U | C | U | G | C | A | U | | C | C | A | G |  |  |  |
| csi-mir162 |  |  |  |  |  |  |  |  |  |  |  |  |  |  |  |  |  | |  |  |  |  |  |  |  |
| mir165 | U | C | G | G | A | C | C | A | G | G | C | U | U | C | A | U | C | | C | C | C |  |  |  |  |
| csi-mir165 |  |  |  |  |  |  |  |  |  |  |  |  |  |  |  |  |  | |  |  |  |  |  |  |  |
| mir166a | U | C | G | G | A | C | C | A | G | G | C | U | U | C | A | U | U | | C | C | C | C |  |  |  |
| csi-mir166a |  |  |  |  |  |  |  |  |  |  |  |  |  |  |  |  |  | |  |  |  |  | C |  |  |
| mir166b | U | C | G | G | A | C | C | A | G | G | C | U | U | C | A | U | U | | C | C | C | C |  |  |  |
| csi-mir166b |  |  |  |  |  |  |  |  |  |  |  |  |  |  |  |  |  | |  |  |  | G |  |  |  |
| mir172a | A | G | A | A | U | C | U | U | G | A | U | G | A | U | G | C | U | | G | C | A | U |  |  |  |
| csi-mir172a |  |  |  |  |  |  |  |  |  |  |  |  |  |  |  |  |  | |  |  |  | A |  |  |  |
| mir390 | A | A | G | C | U | C | A | G | G | A | G | G | G | A | U | A | G | | C | G | C | C |  |  |  |
| csi-mir390 |  |  |  |  |  |  |  |  |  |  |  |  |  |  |  |  |  | |  |  |  |  |  |  |  |
| mir482a | U | C | U | U | C | C | C | U | A | C | U | C | C | U | C | C | C | A | | U | U | C | C |  |  |
| csi-mir482a.2 |  |  |  |  |  |  |  |  |  |  |  |  |  | A |  |  |  |  | |  | G |  |  |  |  |
| mir482b | U | C | U | U | C | C | C | U | A | C | U | C | C | U | C | C | C | A | | U | U | C | C |  |  |
| csi-mir482a.4 |  |  |  |  |  |  |  |  |  |  |  |  |  | C |  |  |  |  | |  | G |  |  |  |  |
| mir530 | U | G | C | A | U | U | U | G | C | A | C | C | U | G | C | A | C | | C | A | U | A |  |  |  |
| csi-mir530 |  |  |  |  |  |  |  |  |  |  | G | G |  |  |  |  | U | |  |  | A | U |  |  |  |
| mir844 | U | U | A | U | A | A | G | C | C | A | U | C | U | U | A | C | U | | A | G | U | U |  |  |  |
| csi-mir844 | C |  |  |  |  |  |  |  |  |  |  |  |  | C |  |  |  | |  |  | G |  |  |  |  |
| mir950 | U | C | A | G | G | U | C | C | U | C | G | G | U | G | G | U | U | | U | A | U |  |  |  |  |
| csi-mir950 |  |  |  |  |  |  |  |  |  |  | A |  |  |  |  |  | C | | C |  |  |  |  |  |  |
| mir1027 | U | U | U | C | U | A | U | C | U | U | C | U | C | U | U | C | C | | A | A | U | C |  |  |  |
| csi-mir1027 |  |  |  |  |  |  |  |  | A |  |  |  | A |  |  |  |  | |  |  |  | G |  |  |  |
| mir1044-3p | U | U | G | U | A | G | U | G | C | A | U | A | U | U | U | G | U | | U | U | U |  |  |  |  |
| csi-mir1044-3p |  |  |  |  |  |  |  |  |  | G |  |  |  |  | G |  |  | | A |  |  |  |  |  |  |
| mir1426 | A | G | A | A | U | C | U | U | G | A | U | G | A | U | G | A | U | | U | A | A | A |  |  |  |
|  | U |  |  |  |  |  |  |  |  |  |  |  |  |  |  |  |  | |  | G |  | U |  |  |  |
