# Supplementary material for: Validation and characterization of Citrus sinensis microRNAs and their target genes
Source: BMC Res Notes. 2012 May 15;5:235. doi: 10.1186/1756-0500-5-235 (PMC3436860; doi:10.1186/1756-0500-5-235)
Supplement: Additional file 4 — Predicted targets for fifteen miRNAs identified in C. sinensis . [file 1756-0500-5-235-S4.doc]

**Table S3**

Predicted targets for fifteen miRNAs identified in *C. sinensis*.

| ID | miRNA(3’-5’)/ mRNA(5’-3’) | Target unigene no.  (number of mismatches) | Target protein | Target function | Conserved gene in other plants (E-score) |
| --- | --- | --- | --- | --- | --- |
| csi-miR160 | 3’ACCGUAUGUCCCUCGGUCCGU 5’ | 29592 (1) | Auxin response factor 10 (ARF10) | Transcription factor (TF) | At2g28350 (1e-162) |
|  | AGGCAUACAGGGAGCCAGGCA |  |  |  |  |
| csi-miR165 (N) | 3’CCCCCUACUUCGGACCAGGCU5’ |  |  |  |  |
|  | UUGGGAUGAAGCCUGGUCCGG | 35004 (3) | Homeo domain leucine zipper (HD-Zip) protein |  |  |
|  | CCGGGAUGAAGCCUGGUCCGG | 31207 (3) | HD-Zip protein | TF | AT4G32880 (1e-142) |
|  | CUGGGAUGAAGCCUG GUCCGG | 10373 (3) | HD-Zip protein | TF | AT5G60690 (1e-122) |
| csi-miR166a/b (R) | 3’ CCCCUUACUUCGGACCAGGCU 5’ |  |  |  |  |
|  | CCGGGAUGAAGCCUGGUCCGG | 31207 (3) | HD-Zip protein | TF | AT4G32880 (1e-142) |
|  | CUGGGAUGAAGCCUG GUCCGG | 10373 (3) | HD-Zip protein | TF | AT5G60690 (1e-122) |
| csi-miR172 (R) | 3’UACGUCGUAGUAGUUCUAAGA5’ |  |  |  |  |
|  | GUGCAGCAUCAUCAGGAUUCU | 24193 (2) | APETALA2- like protein (AP2) | DNA binding / transcription factor (DBF) | AT2G28550 (1e-91) |
| miR390 (N) | 3’CCGCGAUAGGGAGGACUCGAA5’ | No |  |  |  |
| csi-miR482a.2 (R) | 3’ ccguacccaccucaucccuucu 5’ | No |  |  |  |
|  | GGAAUGGGUGGAGUNGGGAAGA | 75213 (2) | disease resistance protein (NBS-LRR class) | defense response | *Populus trichocarpa* |
| csi-miR482a.4 | 3’ ccGuacccCccUCaucccuucu 5’ | No |  |  |  |
|  | GGAAUGGGUGGAGUNGGGAAGA | 75213 (3) | disease resistance protein (NBS-LRR class) | defense response | *Populus trichocarpa* |
| csi-miR530 | 3’ UACUACGUGGACGUUUACGU 5’ | No |  |  |  |
| csi-miR844 | 3’UGGAUCACUCUACCGAAUAUC5’ | No |  |  |  |
| csi-miR950 | 3’UACCUGGUGACUCCUGGACU5’ | No |  |  |  |
| csi-miR1027 | 3’GUAACCUUAUCUACUAUCUUU5’ | No |  |  |  |
| csi-miR1044-3p | 3’UUAUGGUUAUGCGUGAUGUU5’ | No |  |  |  |
| csi-miR1426 | 3’UAGUUAGUAGUAGUUCUAAGU5’ | No |  |  |  |
