# Supplementary material for: Validation and characterization of Citrus sinensis microRNAs and their target genes
Source: BMC Res Notes. 2012 May 15;5:235. doi: 10.1186/1756-0500-5-235 (PMC3436860; doi:10.1186/1756-0500-5-235)
Supplement: Additional file 5 — Expression patterns of 3′ (A) and 5′ (B) products of miRNA cleaved target genes from C. sinensis by RLM-RACE and PPM-RACE. QRT-PCR of HMW RNA isolated from tissues at different development stages. A, B, C, D, E, F, G, H, I, J, K, L, M, and N are the samples of young stems, mature stems, old stems, young leaves, mature leaves, old leaves, flower buds, half open flowers, open flowers, and fruits of different stages (15, 45, 75, 105 and 145 DAFB) Each reaction was repeated three times and the template amount was corrected by 5.8 s rRNAs. [file 1756-0500-5-235-S5.doc]

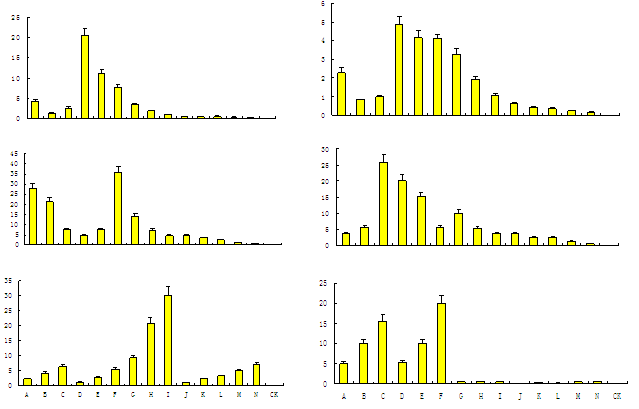


Relative expression

Relative expression

**d** UC52-10373 3’

**f** UC52-75213 3’

A

**e** UC52-24193 3’


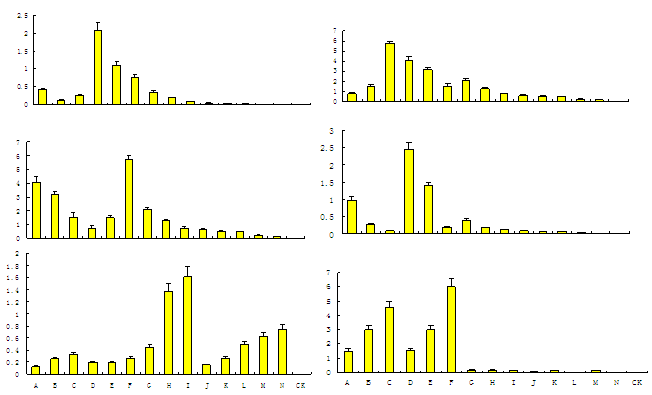


Relative expression

Relative expression

Relative expression

**a** UC52-29592 3’

**b** UC52-35004 3’

**c** UC52-31207 3’

B

Relative expression

Relative expression

Relative expression

Relative expression

Relative expression

Relative expression

**a** UC52-29592 5’

**b** UC52-35004 5’

**c** UC52-31207 5’

**d** UC52-10373 5’

**e** UC52-24193 5’

**f** UC52-75213 5’

**Fig. S2**
