# Supplementary material for: Validation and characterization of Citrus sinensis microRNAs and their target genes
Source: BMC Res Notes. 2012 May 15;5:235. doi: 10.1186/1756-0500-5-235 (PMC3436860; doi:10.1186/1756-0500-5-235)
Supplement: Additional file 6 — The primer sequences of miRNA target genes for QRT-PCR. [file 1756-0500-5-235-S6.doc]

**Table S4**

**The primer sequences of miRNA target genes for qRT-PCR.**

| Target gene | Forward primer sequences (5'-3') | Reverse primer sequences (5'-3') | Amplified  Size (bp) |
| --- | --- | --- | --- |
| UC52-29592 | ACTATGCCGTTATTTTCAGG | TGGATAGAAACAGCCCTGAC | 123 |
| UC52-35004 | TGCAGAGGAGACTTTAACAGAG | CAACCATGAGAAATAGCAACGA | 145 |
| UC52-31207 | TGTCGATTGCAGAGGAGACTTTA | AGGATTTCAGCGACTCTTGTAGG | 160 |
| UC52-10373 | GCAGAGGAGACCTTGGCAGAGTT | AATGGCAAAGATCCCAACCGAAT | 132 |
| UC52-24193 | TTAACTCCAGGCAGCAGCAAGAGA | GGAGGGTCTAACAAGGGAGTGGAAT | 140 |
| UC52-75213 | GTTGGCTTGGACTCCATTATCAG | CGTGCTCTGTATCTCGAAACTTAT | 151 |
