# Supplementary material for: Validation and characterization of Citrus sinensis microRNAs and their target genes
Source: BMC Res Notes. 2012 May 15;5:235. doi: 10.1186/1756-0500-5-235 (PMC3436860; doi:10.1186/1756-0500-5-235)
Supplement: Additional file 7 — The primer sequences of the 5′ products of miRNA cleaved target genes for QRT-PCR. [file 1756-0500-5-235-S7.doc]

**Table S5**

The primer sequences of the 5’ products of miRNA cleaved target genes for qRT-PCR.

| 5’ products of miRNA cleaved target genes | Forward primer sequences (5'-3') | Reverse primer sequences (5'-3') | Amplified product size (bp) |
| --- | --- | --- | --- |
| UC52-29592 | ACTATGCCGTTATTTTCAGG | ATTCTAGAGGCCGAGGCGGCCGACATG | 115 |
| UC52-35004 | TGCAGAGGAGACTTTAACAGAG | ATTCTAGAGGCCGAGGCGGCCGACATG | 120 |
| UC52-31207 | TGTCGATTGCAGAGGAGACTTTA | ATTCTAGAGGCCGAGGCGGCCGACATG | 101 |
| UC52-10373 | GCAGAGGAGACCTTGGCAGAGTT | ATTCTAGAGGCCGAGGCGGCCGACATG | 142 |
| UC52-24193 | TTAACTCCAGGCAGCAGCAAGAGA | ATTCTAGAGGCCGAGGCGGCCGACATG | 115 |
| UC52-75213 | GTTGGCTTGGACTCCATTATCAG | ATTCTAGAGGCCGAGGCGGCCGACATG | 99 |
