# Supplementary material for: Validation and characterization of Citrus sinensis microRNAs and their target genes
Source: BMC Res Notes. 2012 May 15;5:235. doi: 10.1186/1756-0500-5-235 (PMC3436860; doi:10.1186/1756-0500-5-235)
Supplement: Additional file 8 — Primer sequences of the 3′ products of miRNA cleaved target genes for QRT-PCR. [file 1756-0500-5-235-S8.doc]

**Table S6**

**Primer sequences of the 3’ products of miRNA cleaved target genes for qRT-PCR.**

| 3’ products of miRNA cleaved target genes | Forward primer sequences (5'-3') | Reverse primer sequences (5'-3') | Amplified product size (bp) |
| --- | --- | --- | --- |
| UC52-29592 | GGACACTGACATGGACTGAAGGAGT | TGGATAGAAACAGCCCTGAC | 154 |
| UC52-35004 | GGACACTGACATGGACTGAAGGAGT | CAACCATGAGAAATAGCAACGA | 157 |
| UC52-31207 | GGACACTGACATGGACTGAAGGAGT | AGGATTTCAGCGACTCTTGTAGG | 143 |
| UC52-10373 | GGACACTGACATGGACTGAAGGAGT | AATGGCAAAGATCCCAACCGAAT | 145 |
| UC52-24193 | GGACACTGACATGGACTGAAGGAGT | GGAGGGTCTAACAAGGGAGTGGAAT | 147 |
| UC52-75213 | GGACACTGACATGGACTGAAGGAGT | CGTGCTCTGTATCTCGAAACTTAT | 128 |
